# A Quantitative Test of Hamilton's Rule for the Evolution of Altruism

**DOI:** 10.1371/journal.pbio.1000615

**Published:** 2011-05-03

**Authors:** Markus Waibel, Dario Floreano, Laurent Keller

**Affiliations:** 1 Laboratory of Intelligent Systems, School of Engineering, Ecole Polytechnique Fédérale de Lausanne, Lausanne, Switzerland; 2 Department of Ecology and Evolution, Biophore, University of Lausanne, Lausanne, Switzerland; University of Edinburgh, United Kingdom

## Abstract

The evolution of altruism is a fundamental and enduring puzzle in biology. In a seminal paper Hamilton showed that altruism can be selected for when *rb* − *c*>0, where *c* is the fitness cost to the altruist, *b* is the fitness benefit to the beneficiary, and *r* is their genetic relatedness. While many studies have provided qualitative support for Hamilton's rule, quantitative tests have not yet been possible due to the difficulty of quantifying the costs and benefits of helping acts. Here we use a simulated system of foraging robots to experimentally manipulate the costs and benefits of helping and determine the conditions under which altruism evolves. By conducting experimental evolution over hundreds of generations of selection in populations with different *c/b* ratios, we show that Hamilton's rule always accurately predicts the minimum relatedness necessary for altruism to evolve. This high accuracy is remarkable given the presence of pleiotropic and epistatic effects as well as mutations with strong effects on behavior and fitness (effects not directly taken into account in Hamilton's original 1964 rule). In addition to providing the first quantitative test of Hamilton's rule in a system with a complex mapping between genotype and phenotype, these experiments demonstrate the wide applicability of kin selection theory.

## Abstract 2

A study of experimental evolution in simulated groups of foraging robots demonstrates that their propensity to behave altruistically depends on their genetic relatedness (similarity), and the costs and benefits associated with altruistic behavior.

## Author Summary

One of the enduring puzzles in biology and the social sciences is the origin and persistence of altruism, whereby a behavior benefiting another individual incurs a direct cost for the individual performing the altruistic action. This apparent paradox was resolved by Hamilton's theory, known as kin selection, which states that individuals can transmit copies of their own genes not only directly through their own reproduction but also indirectly by favoring the reproduction of kin, such as siblings or cousins. While many studies have provided qualitative support for kin selection theory, quantitative tests have not yet been possible due to the difficulty of quantifying the costs and benefits of helping acts. In this study, we conduct simulations with the help of a simulated system of foraging robots to manipulate the costs and benefits of altruism and determine the conditions under which altruism evolves. By conducting experimental evolution over hundreds of generations of selection in populations with different costs and benefits of altruistic behavior, we show that kin selection theory always accurately predicts the minimum relatedness necessary for altruism to evolve. This high accuracy is remarkable given the presence of pleiotropic and epistatic effects, as well as mutations with strong effects on behavior and fitness. In addition to providing a quantitative test of kin selection theory in a system with a complex mapping between genotype and phenotype, this study reveals that a fundamental principle of natural selection also applies to synthetic organisms when these have heritable properties.

## Introduction

One of the enduring puzzles in biology and the social sciences is the origin and persistence of altruism, whereby a behavior benefiting another individual incurs a direct cost for the individual performing the altruistic action. A solution to this apparent paradox was first provided by Hamilton [1], who showed that a behavior increases in frequency when *rb* − *c*>0, where *c* is the fitness cost to the altruist, *b* is the fitness benefit to the beneficiary, and *r* is their genetic relatedness. While this rule has provided an important framework in which to conceptualize social evolution [2]–[12], it is based on several assumptions, including weak selection, additivity of costs and benefits of fitness components, and a special definition of relatedness that uses statistical correlations among individuals rather than genealogy to describe similarity. Several studies investigated how violations to these assumptions may lead to failures of Hamilton's original 1964 rule [13]–[21], but it is yet unclear how the combined effects of these factors may affect the evolution of altruism in organisms with a complex mapping between genotype and phenotype. It also remains to be investigated to what extent Hamilton's original 1964 rule is influenced by factors such as drift and interactions between loci within genomes [22],[23].

To investigate how a complex mapping between genotype and phenotype can affect the course of social evolution, we conducted artificial evolution with groups of robots in simulations by modifying a system recently developed to investigate the evolution of cooperative transport [24]. Eight small (2×2×4 cm) Alice robots [25] and eight food items were placed in a foraging arena with one white wall and three black walls. The performance of robots was proportional to the number of food items successfully transported to the white wall and the robots were given the option to allocate the fitness rewards of successfully transported items to themselves (selfish behavior) or share them with other group members (altruistic behavior—in this case the fitness reward of the food item was shared equally between the seven other robots in the group). By choosing appropriate fitness values for shared and non-shared food items (see Materials and Methods), it was possible to precisely manipulate the benefits and cost of helping behavior (i.e., the *c* and *b* values of Hamilton's rule, see Materials and Methods).

**Figure 1:**
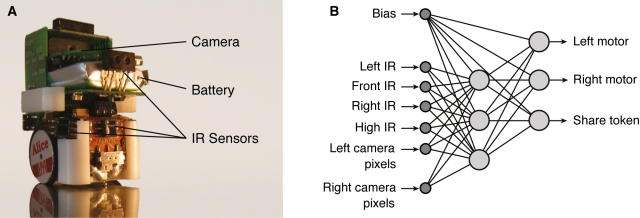
Physical robots and neural network controller. (A) The Alice robots used in the experiments were equipped with infrared distance sensors (IR) and vision sensors (camera). (B) The input neurons (small circles) of the artificial neural network were connected to internal and output neurons (large circles) by 33 connection weights (lines connecting circles).

The robots were equipped with two motorized wheels and three infrared distance sensors that could detect food items up to 3 cm away, a fourth infrared distance sensor with 6 cm range allowing to distinguish food items from robots, and two vision sensors mounted on top of the robot to perceive the color of the arena walls (Figure 1A). These six sensors were connected to a neural network comprising six input neurons, three hidden neurons, and three output neurons (Figure 1B). Two output neurons determined the speeds of the wheels, while the third neuron determined whether the food items successfully collected were shared or not. The genome of the robots (33 genes) encoded the 33 connection weights of the neural network (see Materials and Methods) and thus determined how sensory information was processed and how robots behaved. Our analyses reveal that this system resulted in both pleotropic and epistastatic effects as well as a high proportion of mutations having strong effects on behavioral traits (i.e., leading to deviations from the assumption of weak selection).

## Results

We conducted 500 generations of selection in a population consisting of 200 groups. The probability of robots to transmit their genomes from one generation to the next was proportional to their individual fitness (see Materials and Methods). The selected genomes were randomly assorted and subjected to crossovers and mutations to create the 1,600 new genomes (200 groups of 8 robots) forming the next generation [24].

This experimental setup allowed us to independently manipulate the relatedness between robots within a group and the cost-to-benefit ratios of helping. To quantitatively test Hamilton's rule for the evolution of altruism, we investigated how the level of altruism (defined as the proportion of food items shared with other group members) changed over generations in populations with five different *c/b* ratios and five relatedness values (see Materials and Methods). For each of these 25 treatments, the selection experiments were conducted in 20 independently evolving populations. Because of the impossibility to conduct hundreds of generations of selection with real robots, we used physics-based simulations that precisely model the dynamical and physical properties of the robots. We previously showed that evolved genomes can be successfully implemented in real robots [26] that display similar behavior to that observed in the simulations.

**Figure 2:**
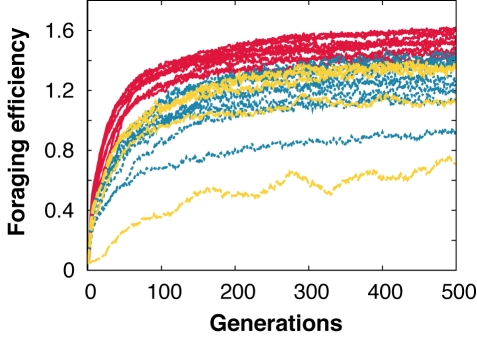
Mean group foraging efficiency during the 500 generations of selection. Lines represent the mean number of items transported successfully for each of the 25 treatments (five relatedness values × five *c/b* ratios, 20 replicates per treatment), with yellow dashed lines representing treatments where *r  =  c/b*, red lines treatments where *r* < *c/b*, and blue dotted lines treatments where *r* > *c/b*.

**Figure 3:**
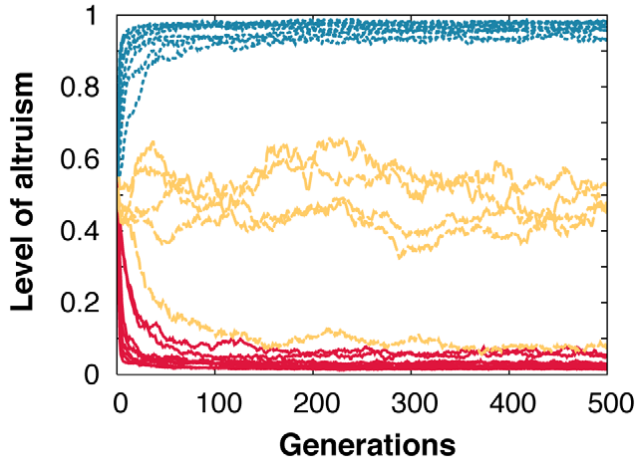
Mean level of altruism during the 500 generations of selection. Lines represent the mean level of altruism, defined as the proportion of food items shared with other group members, for each of the 25 treatments (five relatedness values × five *c/b* ratios, 20 replicates per treatment), with yellow dashed lines representing treatments where *r  =  c/b*, red lines treatments where *r* < *c/b*, and blue dotted lines treatments where *r* > *c/b*.

**Figure 4:**
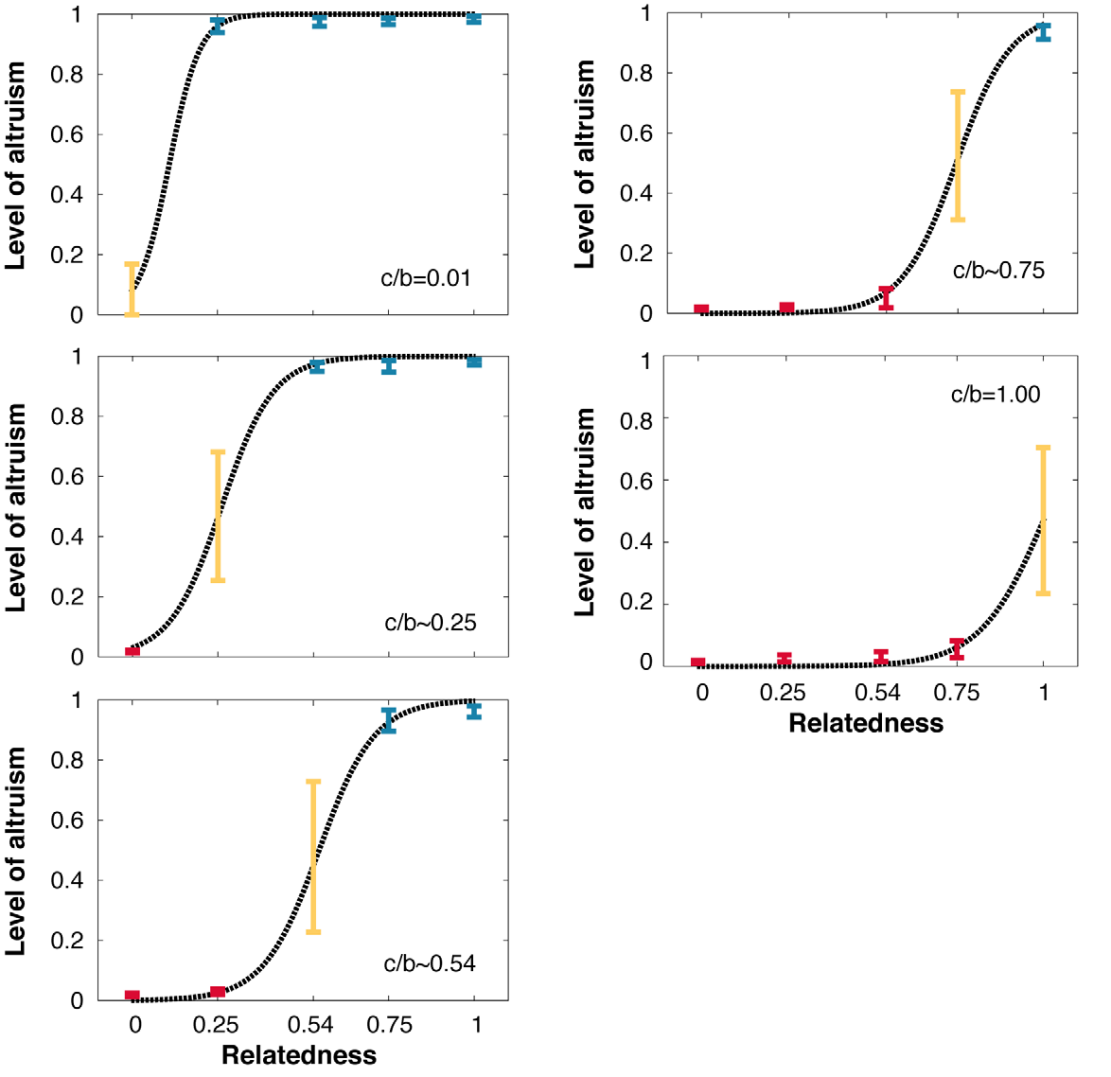
Mean level of altruism at the end of the 500 generations of selection. The mean (±SD) level of altruism as a function of the *c/b* ratio and level of relatedness (20 replicates per treatment) for *r  =  c/b* (yellow lines), *r* < *c/b* (red lines), and *r* > *c/b* (blue lines). Dashed lines are fits based on a model by McNamara et al. [44]–[46].

Because the 33 genes were initially set to random values, the robots' behaviors were completely arbitrary in the first generation. However, the robots' performance rapidly increased over the 500 generations of selection (Figure 2). The level of altruism also rapidly changed over generations with the final stable level of altruism varying greatly depending on the within-group relatedness and *c/b* ratio (Figure 3). When the *c/b* value was very small (0.01), the level of altruism was very high in the populations where within-group relatedness was positive (i.e., 0.25, 0.5, 0.75, and 1.00) and close to zero when robots were unrelated (Figure 4). In the treatments with other *c/b* values, the level of altruism was also very low when the relatedness was close to 0 and the level of altruism also rapidly increased when the relatedness became higher than a given value. In all cases, the transition occurred when *r* became greater than *c/b*, as predicted by Hamilton's rule.

When the relatedness was equal to *c/b*, there was an intermediate level of altruism with the frequency of altruistic acts not differing significantly from the initial value, which was 0.5 (four one-sample Wilcoxon tests, *df* = 19, all *p>*0.368). This is the expected pattern because the inclusive fitness of robots, comprising both their own fitness points and those gained from altruists, is independent of whether or not they behave altruistically when *r  =  c/b*. Under such conditions, the level of altruism should vary only as a result of drift over generations, thus leading to important between-population variation in the level of altruism. Consistent with this prediction, the standardized variance (F  =  Var(p)/pq) in altruism when *r* was equal to *c/b* (*F* = 0.204) was significantly higher than when *r* was greater than *c/b* (*F* = 0.018; Mann-Whitney, *df* = 13, *p = *0.002) and when *r* was smaller than *c/b* (*F* = 0.015; Mann-Whitney, *df* = 13, *p<*0.003).

The fact that the level of altruism remained slightly greater than 0 when *r* was smaller than *c/b* and slightly lower than 1 when *r* was greater than *c/b* can be explained by mutations maintaining some behavioral variability in the population. In line with this view of the level of altruism being at mutation-selection equilibrium, the level of altruism became significantly closer to zero (Pearson's *r = *0.643; Mann-Whitney, *df* = 13, *p<*0.001) as the strength of selection increased (i.e., when the value *r* − *c/b* became more negative, only negative values of *r* − *c/b* considered for the correlation). Similarly, the level of altruism became significantly closer to 1 (Pearson's *r = *0.805; Mann-Whitney, *df* = 13, *p<*0.004) as the strength of selection for higher levels of altruism increased (i.e., when the value *r* − *c/b* increased, only positive values of *r* − *c/b* considered in the correlation).

To determine whether mutations in our neural network had pleiotropic and epistatic effects and whether there were departures from weak mutations effects, we conducted additional experiments at the last generation in two treatments with intermediate *r* and *c/b* values (treatment 1: *r = *0.25, *c/b* = 0.75; treatment 2: *r = *0.75, *c/b* = 0.25). First, for each treatment, we subjected 4,000 individuals (one in each group) to a single mutation of moderate effect (see Materials and Methods). In the first experiment, performance was significantly affected by a much higher proportion of the mutations than the level of altruism (Table 1). Importantly, 1.36% of the mutations affecting the level of altruism also translated into a significant change in performance, indicating widespread pleiotropic effects. Similar results were obtained in the second experiment with 4.91% of the mutations affecting the level of altruism also significantly affecting performance. Second, we tested for epistatic effects by comparing the effect of a single mutation in 4,000 individuals with two allelic variants at another locus (see Materials and Methods). The genetic background significantly influenced the effect of the mutation in 2,371 (59.3%) of the cases in the first treatment and 2,336 (58.4%) of the cases in the second treatment. These results demonstrate that epistatic interactions are also widespread. Finally, our experiments showed frequent departures from weak effects on behavior and fitness. Performance changed by more than 25% for 1,616 (40.4%) of the mutations in the first treatment and 1,776 (44.4%) of the mutations in the second treatment, and the level of altruism changed by more than 25% for 552 (13.8%) and 1,808 (45.2%) of the mutations in the first and second treatment, respectively.

**Table 1:** Effects of a single mutation on performance, altruism, and both.

Treatment	Performance	Altruism	Both
*r* = 0.25, *c*/*b* = 0.75	2,524	86	54
*r* = 0.75, *c*/*b* = 0.25	376	243	189

Number of individuals for whom a single mutation of moderate effect significantly altered only performance, only the level of altruism, and both performance and the level of altruism (i.e., pleiotropic effects). Experiments were performed on 4,000 individuals in the two treatments with intermediate *r* and *c/b* values (see Materials and Methods).

## Discussion

Although Hamilton's original 1964 rule provides a general framework of how natural selection works [17],[27], its theoretical and empirical applications usually involve the limiting assumptions of weak selection and additivity of costs and benefits of fitness components as well as the absence of pleiotropic and epistatic gene interactions [15],[16],[28] (but see [13] for relaxations of some of these assumptions in concrete applications), leading to the conclusion that the *rb* − *c*>0 rule should be used with caution when there are pleiotropic, epistatic, and non-additive effects [29],[30]. Interestingly, the genetic architecture of the robots in our system also led to departure from all these assumptions with the exception of non-additivity of costs and benefits of fitness components. However, the occurrence of non-additive (epistatic) effects of mutations at several loci in the genome leads to a situation that is conceptually similar to non-additivity of costs and benefits of fitness components [22]. In both cases, the fitness depends non-additively on gene action, with the interaction involving alleles at two loci on the same genome in the case of non-additive (epistatic) gene effects, and alleles at two homologous loci on two different genomes in the case of non-additivity of costs and benefits of fitness components.

Despite the fact that the assumptions mentioned above were not fulfilled, Hamilton's original 1964 rule always accurately predicted the conditions under which altruism evolved in our system. Whatever the *c/b* value used, altruism always evolved in populations where *r* was greater than *c/b*. This finding is important given that the assumption of weak selection, additivity of costs and benefits of fitness components and absence of pleiotropic and epistatic gene interactions are also likely to be violated in real organisms that also have a complex mapping between genomes and phenotypes.

Another important issue relates to the measure of relatedness. There has been considerable confusion in the literature since relatedness coefficients actually measure more than pedigree coefficients and because different derivations of Hamilton's rule take as their focal trait a variety of different quantities [16],[17],[30]. In the original derivation of Hamilton's rule [1] and many that followed (e.g., [12],[31]), the trait of interest was the genetic value at a single gene position and the regression coefficient of relatedness corresponded to an identity in state relative to the population average [31]. The interest in social evolution where social partners tend to be genealogical kin [1] has led to the use of Wright's *F* statistics as a measure of relatedness (e.g. [12],[22],[32]). Alternatively, Hamilton's rule has been derived to express the change in the social behavior phenotype (e.g., [16],[22],[33],[34]), often considered as a quantitative trait with many underlying gene positions contributing. In this case the coefficient of relatedness represents a regression of some measure of the individual's genetic value for that trait such as a breeding value [17], *p* score [16], gene frequency [1],[12], or partner phenotype on its own phenotype value [34].

Interestingly, the simple genetic structure of our groups leads to all these measures of relatedness being identical. In all our experiments groups were started by individuals randomly chosen from the previous generations. The relatedness between these founding individuals is therefore zero as they are not more genetically or phenotypically similar within groups than between groups. Positive within-group relatedness was created by cloning the founding individuals. Thus, positive relatedness was only due to one-generation coancestry and the probability that benefits of altruism being provided to a clone compared to an unrelated individual. Such a breeding system is conceptually very similar to that Hamilton had in mind when trying to explain the evolution of reproductive altruism in social insects where the sterile (altruistic) workers are the offspring of their mother queen (the individual benefitting from the altruistic worker behavior). The relatedness in such a system can also be described in terms of identity by descent [35], which provides an approximation of identity in state for rare genetic variants (see [31] for a recent review). Of interest would be to test in future studies how the evolution of altruism is influenced by more complex population structures where the effect of strong selection may lead to variation in within-genome differences in the covariance between genes in different individuals.

Because the rewards provided by the food items were either assigned to the focal individual who successfully transported it (selfish behavior) or shared equally between all the other group members (altruistic behavior), the fitness effects were additive and there were no synergetic effects. Thus, the cost incurred by an individual sharing altruistically a food item and the benefits to the other group members was not dependent on the recipients' genotypes and the proportion of them being altruistic. The lack of such synergetic effects results in the costs and benefits associated with an altruistic act being independent of the genotypic composition of the groups and the overall level of altruism in the population (i.e., there are no frequency-dependent effects). In natural systems there are frequently synergetic effects and this is one of the main reasons why it is not possible to reliably quantify the cost and benefits associated with altruistic actions (e.g., [15],[16],[36],[37]).

From an empirical perspective, our study is therefore valuable because there have been many tests of Hamilton's rule, but these studies are usually not quantitative due to the impossibility of assessing the costs and benefits of altruistic acts, even in the most simple social systems such as those documented in some bacteria [10],[38], social amoebae [39], or even synthetic microbial systems [36]. Our study also demonstrates that contrary to some misunderstandings [3], kin selection does not require specific genes devoted to encode altruism or sophisticated cognitive abilities, as the neuronal network of our robots comprised only 33 neurons. More generally, this study reveals that a fundamental principle of natural selection also applies to synthetic organisms when these have heritable properties [40].

## Materials and Methods

### Experimental Setup, Robots, and Neural Architecture

Groups of eight Alice micro-robots and eight food items were placed into a 50×50 cm foraging arena. We chose a collective foraging task to investigate the evolution of altruism, because foraging efficiency is a key factor for many biological social groups such as ant or bee colonies [41]. Foraging required robots to locate a food item, to position themselves in front of the item, and to push it into a 4-cm-wide target zone along the white wall of the arena (the three other walls were black).

Robots were controlled by a feed-forward neural network consisting of six sensory input neurons, one bias input neuron, and six neurons with sigmoid activation. The robots had four infrared distance sensors, three of them sensing objects within a 3 cm range and the fourth, which was placed higher, having a 6 cm range. These sensors allowed robots to locate the food items and distinguish them from robots. Robots were also equipped with two vision sensors to see the white wall [24].

These six sensory inputs were scaled to a range of [−1; 1]. In addition to the sensory inputs the neural network also comprised a bias input set to a constant value of −1, which was used to encode the neuron firing threshold. These seven inputs were connected to three neurons in a hidden layer, which in turn connected to three output neurons. The strength of these 33 connections was determined by 33 genes, whose values ranged from 0 to 255 (i.e., 8 bit resolution per gene). The activation of each of the six hidden and three output neurons was calculated by multiplying each of its input values by its associated connection weight, summing over all inputs, and passing the sum through the continuous tanh(x) function to obtain the neuron's activation value in the range of [−1; 1]. The activation value of the first output neuron controlled the left motor speed, the second the right motor speed, and the third whether or not the successfully pushed food items were shared with other group members.

### Relatedness, *c/b* Ratio, and Artificial Evolution

We used five different levels of relatedness in the experiments. To create groups of unrelated individuals (r  = 0), we randomly distributed the 1,600 individuals in the 200 groups. To obtain groups with a relatedness of r  =  1, we cloned one individual 7 times and formed groups with 8 genetically identical individuals. To create groups with a relatedness of approximately r =  0.75, we used two individuals (A and B) and cloned one seven times (clone proportion A:B = 1:7). The resulting relatedness in these groups was thus r,0.7492. To create groups with a relatedness close to r  =  0.5, we similarly composed each group of three types of clones but in proportions 6:1:1, which led to r,0.5357. To create groups with a relatedness close to r =  0.25, we again composed each group of three types of clones, but this time using proportions 3:3:2, which resulted in a relatedness of r,0.2468. The genetic composition of groups thus differed from that of most animal groups in that some individuals were clones (*r = *1) rather than belonging to kin classes such as full siblings (*r = *0.5) or cousins (*r = *0.125). However, in the absence of preferential interactions between kin, social evolution should be influenced by the average group relatedness. This is because genetic relatedness depends on interaction probabilities of genes [4], which in our model is equivalent to interaction probabilities between clonal individuals. Our experimental setup prevented preferential interactions between individuals by randomizing starting positions, having all robots being identical, and using a neural network that did not allow individuals to memorize past interactions.

To manipulate the *c* and *b* value of Hamilton's rule we modified the fitness values for shared and non-shared food items that were successfully transported. When non-shared, a food item provided a reward *c* to the selfish individual. When shared, the food item provided no direct benefit to the focal individual but a benefit *b* equally shared by the seven other robots in the group. The *c/b* ratios used were calculated using Queller's approach [42]. We used a value of 0.01 for the smallest *c/b* ratio because with a value of *c/b* = 0, there is no selection for foraging efficiency when *r = *0, hence resulting in many populations going extinct (because no items were successfully foraged).

The foraging efficiency of each group was evaluated 10 times for 60 seconds and the inclusive fitness of each individual was estimated according to the number of food items collected and not shared + the number of food items that other group members collected and shared (these values being multiplied by *c* and *b*/7, respectively). The probability of the genome of a given robot to contribute to the next generation was directly proportional to the robot's inclusive fitness (roulette wheel selection with replacement [43]). Selected genomes were paired to conduct a crossing over with a probability of 0.005. The resulting genomes were subjected to mutation (probability of 0.005 per bit; i.e., 0.04 per gene). This process of selection, recombination, and mutation was repeated until there were enough genomes for the 1,600 individuals (200 groups) of the next generation. The level of altruism was calculated for each group as the proportion of collected food items that was shared within a group: *A*  =  *n*(*a*)/(*n*(*a*) + *n*(*s*)), where *n*(*a*) was the number of collected food items individuals shared and *n*(*s*) the number of items individuals did not share.

All 25 selection experiments were repeated 20 times (20 independent replicates). Evolution lasted for 500 generations for each experimental condition. For statistic analyses, the fitness and the level of altruism of all 200 groups in each of the 20 replicates were averaged over the last 10 generations. Means were compared with Mann-Whitney tests as Shapiro-Wilk tests showed that in many treatments the data did not follow a normal distribution.

### Calculating Relatedness, Costs, and Benefits

In each of the individually evolving populations, altruistic interactions always occurred within groups, while the reproductive competition occurred at the level of the population. To manipulate the relatedness, we cloned genomes for each group and formed groups of different proportions of clones. Each group was composed of *k* different types of clones with respective frequencies *x_i_*, *i*  = 1 … *k*, 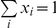.

The genetic relatedness *r* quantifies the greater (or smaller) genetic similarity between individuals compared to the population average. Using the regression definition of relatedness [16],[42],
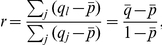
where *j* indexes the individuals in the population and *l* indexes the social partner of *j*.

In our system 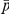 corresponds to the average probability of a focal individual being genetically identical to another member of the population and 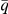 to the average probability of a focal individual being a genetically identical clone of another member of its group. Assuming that populations contain *m* groups with *n* individuals each,
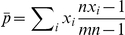


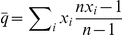



In all experiments the independently evolving populations consisted of *m* = 200 groups, each composed of *n* = 8 individuals.

Given that the evolution of social behavior is influenced by the relative rather than the absolute values of costs and benefits, we arbitrarily set 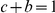 and calculated the costs *c* and benefits *b* for the expected transition from selfish to altruistic behavior as
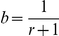


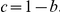



### Pleiotropic Effects

To test for pleiotropic effects, we studied the outcome of a single mutation on two behavioral measures, performance and altruism. Performance was determined as the number of food items collected by an individual, and the level of altruism as the percentage of these food items shared with other group members. One mutation was performed on one individual in each of the 200 groups for each of the 20 replicates at the last generation for each of two treatments with intermediate values of relatedness and *c/b* ratio (treatment 1∶ *r = *0.25, *c/b* = 0.75; treatment 2∶ *r = *0.75, *c/b*  = 0.25). All 8,000 individuals were subjected to a mutation of medium effect. This was achieved by flipping, for each individual, the third of the eight bits of a randomly chosen gene, hence always resulting in a mutation size ±32. We chose this value because it was the median value of the mutations (range ±128) the robots were subjected to in the 500 generations of selection. The performance and level of altruism of each mutated individual was then evaluated in 100 independent trials in its group and compared to its performance and level of altruism before the mutation (Wilcoxon rank sum tests using a 5% significance level).

For the first treatment (*r  = *0.25, *c/b* = 0.75), rank sum tests could be conducted for 3,961 out of the 4,000 individuals as 39 individuals did not collect any food item either before or after the mutation, hence preventing determination of the level of altruism. For the second treatment (*r* = 0.75, *c/b* = 0.25), rank sum tests could be conducted for 3,848 out of the 4,000 individuals, as 152 individuals did not collect any food item either before or after the mutation.

### Epistatic Effects

To test for epistatic effects, we used the same individuals as used in the experiment on pleiotropic effects and assessed the performances of individuals without a mutation *F*(0) and with a mutation *F*(A). We then subjected each of these 16,000 individuals (8,000 without and 8,000 with a mutation) to a new mutation B (also of median effect) and assessed their fitnesses *F*(B) and *F*(AB). We then compared whether this new mutation had a similar effect on the fitness of individuals with and without the first mutation by evaluating each of the resulting 32,000 individuals in 100 independent trials and calculating *z* scores based on the standard deviation (SD) and mean fitness 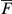


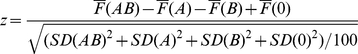



*Z* scores could be calculated for 3.998 and 3,978 out of the 4,000 individuals for the first and second treatment, respectively (2 and 22 individuals, respectively, did not collect any food items). Statistics used a 5% (*z* = 2) significance level.

### Weak Selection

Models of social evolution, as most models in evolutionary biology, usually resort to weak selection, where different individuals have very similar fitness. To test whether the mutations frequently had large effects (i.e., whether there was departure from weak selection), we determined how frequently a mutation of median effect resulted in a greater than 25% change in performance and the level of altruism (4,000 individuals per treatment). Note that the value of 25% was arbitrarily chosen as there is no convention of what change in fitness can be assumed to be a departure of weak selection. Again Wilcoxon rank sum tests were performed on the 100 trials per individual with a 5% significance level.

## References

[1] Hamilton W. D (1964). The genetical evolution of social behavior I+II.. J Theor Biol.

[2] Foster K. R, Wenseleers T, Ratnieks L. W (2006). Kin selection is the key to altruism.. Trends Ecol Evol.

[3] Dawkins R (1979). Twelve misunderstandings of kin selection.. Z Tierpsychol.

[4] Bourke A. F. G, Franks N. R (1995). Social evolution in ants..

[5] Lehmann L, Keller L (2006). The evolution of cooperation and altruism: A general framework and a classification of models.. J Evol Biol.

[6] Sundström L, Chapuisat M, Keller L (1996). Conditional manipulation of sex ratios by ant workers: a test of kin selection theory.. Science.

[7] Pfennig D. W, Collins J. P (1993). Kinship affects morphogenesis in cannibalistic salamanders.. Nature.

[8] West S. A, Murray M. G, Machado C. A, Griffin A. S, Herre E. A (2001). Testing Hamilton's rule with competition between relatives.. Nature.

[9] Griffin A. S, West S. A (2003). Kin discrimination and the benefit of helping in cooperatively breeding vertebrates.. Science.

[10] Griffin A. S, West S. A, Buckling A (2004). Cooperation and competition in pathogenic bacteria.. Nature.

[11] Hamilton W. D (1972). Altruism and related phenomena, mainly in social insects.. Ann Rev Ecol Syst.

[12] Hamilton W. D (1970). Selfish and spiteful behaviour in an evolutionary model.. Nature.

[13] Roze D, Rousset F (2008). Multilocus models in the infinite island model of population structure.. Theor Popul Biol.

[14] Gardner A, Hardy I. C, Taylor P. D, West S. A (2007). Spiteful soldiers and sex ratio conflict in polyembryonic parasitoid wasps.. Am Nat.

[15] Michod R. E (1982). The theory of kin selection.. Ann Rev Ecol Syst.

[16] Grafen A (1985). A geometric view of relatedness.. Oxf Surv Evol Biol.

[17] Queller D. C (1992). A general model for kin selection.. Evolution.

[18] Karlin S, Matessi C (1983). Kin selection and altruism.. Proc R Soc London B.

[19] Charlesworth B (1978). Some models of the evolution of altruistic behavior between siblings.. J Theor Biol.

[20] Uyenoyama M. K, Feldman M. W (1980). Theories of kin and group selection: a population genetics perspective.. Theor Popul Biol.

[21] Nowak M. A, Tarnita C. E, Wilson E. O (2010). The evolution of eusociality.. Nature.

[22] Frank S. A (1998). Foundations of social evolution..

[23] Lehmann L, Rousset F (2009). Perturbation expansions of multilocus fixation probabilities for frequency-dependent selection with applications to the Hill-Robertson effect and to the joint evolution of helping and punishment.. Theor Popul Biol.

[24] Waibel M, Keller L, Floreano D (2009). Genetic team composition and level of selection in the evolution of cooperation.. IEEE Trans Evol Comput.

[25] Caprari G, Estier T, Siegwart R (2001). Fascination of down scaling - Alice the sugar cube robot.. J Microm.

[26] Floreano D, Mitri S, Magnenat S, Keller L (2007). Evolutionary conditions for the emergence of communication in robots.. Curr Biol.

[27] Frank S. A (1997). The Price equation, Fisher's fundamental theorem, kin selection, and causal analysis.. Evolution.

[28] Seger J (1981). Kinship and covariance.. J Theor Biol.

[29] Cavalli-Sforza L, Feldman M (1978). Darwinian selection and "altruism.. " Theor Popul Biol.

[30] Gardner A, West S. A, Barton N. H (2007). The relation between multilocus population genetics and social evolution theory.. Am Nat.

[31] Roze D, Rousset F (2004). The robustness of Hamilton's rule with inbreeding and dominance: kin selection and fixation probabilities under partial sib mating.. Am Nat.

[32] Michod R. E, Hamilton W. D (1980). Coefficients of relatedness in sociobiology.. Nature.

[33] Queller D. C (1992). Does population viscosity promote kin selection?. Trends Ecol Evol.

[34] Queller D. C (1985). Kinship, reciprocity, and synergism in the evolution of social behaviour.. Nature.

[35] Malécot G (1948). The mathematics of heredity (translated from the French edition, 1948): Freeman & Co.

[36] Chuang J. S, Rivoire O, Leibler S (2009). Simpson's paradox in a synthetic microbial system.. Science.

[37] Gore J, Youk H, van Oudenaarden A (2009). Snowdrift game dynamics and facultative cheating in yeast.. Nature.

[38] Rainey P, Rainey K (2003). Evolution of cooperation and conflict in experimental bacterial populations.. Nature.

[39] Strassmann J. E, Zhu Y, Queller D. C (2000). Altruism and social cheating in the social amoeba dictyostelium discoideum.. Nature.

[40] Maynard Smith J, Szathmary E (1995). The major transitions in evolution..

[41] Seeley T. D (1995). The wisdom of the hive: the social physiology of honey bee colonies..

[42] Queller D. C (1994). Genetic relatedness in viscous populations.. Evol Ecol.

[43] Back T (1996). Evolutionary algorithms in theory and practice: evolution strategies, evolutionary programming, genetic algorithms..

[44] McNamara J. M, Webb J. N, Collins E, Szekely T, Houston A. I (1997). A general technique for computing evolutionarily stable strategies based on errors in decision-making.. J Theor Biol.

[45] Kokko H (2003). Are reproductive skew models evolutionarily stable?. Proc R Soc Biol Sci Ser B.

[46] Gardner A, West S. A (2004). Cooperation and punishment, especially in humans.. Am Nat.

